# Photonics of Hydrothermally Treated β‐Lactoglobulin Amyloids

**DOI:** 10.1002/smsc.202400054

**Published:** 2024-04-24

**Authors:** Piotr Hanczyc, Serena Rosa Alfarano, Sreenath Bolisetty, Jiangtao Zhou, Mohammad Peydayesh, Viviane Lutz‐Bueno, Ana Diaz, Shrestha Roy Goswami, Maarten T. P. Beerepoot, Mohammad Mehboob Alam, Lei Wang, Niclas Solin, Iwona Szymanska, Raffaele Mezzenga

**Affiliations:** ^1^ Faculty of Physics Institute of Experimental Physics University of Warsaw Pasteura 5 02‐093 Warsaw Poland; ^2^ Department of Physics, Chemistry, and Biology, Electronic and Photonic Materials, Biomolecular and Organic Electronics Linköping University 581 83 Linköping Sweden; ^3^ Department of Health Sciences and Technology ETH Zurich 8092 Zurich Switzerland; ^4^ Paul Scherrer Institut PSI 5232 Villigen Switzerland; ^5^ Hylleraas Centre for Quantum Molecular Sciences Department of Chemistry UiT The Arctic University of Norway N‐9037 Tromsø Norway; ^6^ Department of Chemisry and Department of Materials Science and Metallurgical Engineering Indian Institute of Technology Bhilai Durg Chhattisgarh 491002 India; ^7^ School of Chemical Engineering Guangdong University of Petrochemical Technology Maoming Guangdong 525000 China; ^8^ Department of Food Technology and Assessment Institute of Food Sciences Warsaw University of Life Sciences (WULS‐SGGW) 159C Nowoursynowska St. 02‐776 Warsaw Poland; ^9^ Department of Materials ETH Zurich 8093 Zurich Switzerland

**Keywords:** amyloids, enhanced emission, hydrothermal treatment, Light emitting diode (LED), nonlinear absorption

## Abstract

Increased temperature and high pressure are applied to β‐lactoglobulin fibrils in the autoclave, resulting in the acquisition of a composite material comprised of partially disassembled amyloid fibrils and carbon dots. Confirmation of the preservation of the β‐sheet motif attributed to amyloids in the hydrothermally treated fibrils is obtained through wide‐angle X‐ray scattering and ThT assay. *Z*‐scan analysis reveals a two‐photon absorption (2PA) enhancement in the low‐lying transition band (*L*_a_) of tyrosine, while quantum chemical calculations demonstrate a correlation between the yield of 2PA and the interspace distance between aromatic residues. Overall, the intrinsic optical properties of amyloid fibrils treated in a subcritical water environment are found to be linked with the π‐conjugation of tyrosine units and their through‐space coupling. The resulting composite material is employed as a coating for a commercial ultraviolet light‐emitting diode lamp, showcasing the potential utility of sustainable biomaterials with improved optical properties for photonics applications.

## Graphical abstract

By subjecting β‐lactoglobulin fibrils to elevated temperature and pressure in autoclave, partially disassembled fibrils are generated. The study reveals a correlation between fluorescence and two‐photon absorption and the spacing of aromatic residues, shedding light on the mechanism behind the improved optical properties of amyloid fibrils. Furthermore, the hydrothermally treated β‐lactoglobulin fibrils are utilized to coat ultraviolet light‐emitting diode lamps.

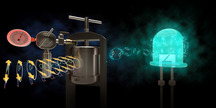

## 1 Introduction

Under appropriate conditions, both, in vivo and in vitro,^[^ ^1^ ^]^ protein molecules self‐assemble into ordered fibrillar structures known as amyloid fibrils. These fibrils can be generated from a wide range of proteins by simply heating the acidic solution of the protein.^[^ ^2^ ^]^ Amyloid fibrils exhibit a high degree of structural regularity, with β‐strands (formed from individual protein molecules) oriented perpendicular to the long axis of the fibril. The inter‐strand distance between β‐strands is ≈4.6 Å, while the inter‐sheet distance between β‐sheets is about 10 Å.^[^ ^3^ ^]^ Individual fibrils can have a length of several micrometers with a typical diameter of 8–10 nm.^[^ ^4^ ^]^

Amyloid fibrils showcase various intriguing emergent optical properties, including nonlinear absorption, light refraction,^[^ ^5^ ^]^ and blue‐green fluorescence.^[^ ^6^ ^]^ Despite these properties, the mechanism behind the optical behavior of fibrils remains elusive, and controlling the yield of nonlinear absorption or fluorescence is challenging due to the high structural complexity of fibrils. This complexity poses a challenge for utilizing fibrils in photonic application.^[^ ^7^ ^]^

Herein, we demonstrate that the optical properties of β‐lactoglobulin fibrils can be modified through additional hydrothermal processing (HTP) using an autoclave at elevated pressure and temperature that keeps water in a liquid phase, resulting in a material with enhanced photonic properties. The study introduces HTP as an alternative to traditional chemical or genetic modifications of proteins to alter their structure.^[^ ^8^ ^]^ In the context of progressing number of reports on blue‐emitting materials, a field that has seen significant interest due to their applications in imaging,^[^ ^9^ ^]^ sensing,^[^ ^10^ ^]^ and light‐emitting devices,^[^ ^11^ ^]^ this study introduces affordable amyloid fibrils as a starting material for hydrothermal modifications. Unlike standard approaches whereby undefined biomass or waste materials become converted in a hydrothermal process into blue‐emitting carbon dots, fibrils have a predefined structure before processing. Herein, in a comprehensive analysis, hydrothermally treated fibrils were demonstrated to show unique properties correlated with their structural rearrangement. In a subcritical water environment, re‐polymerization and aromatization reactions cause modification of the intrinsic structure of β‐lactoglobulin fibrils, leading to enhanced optical properties. This study contains comprehensive experimental dataset on hydrothermally treated fibrils fluorescence and two‐photon absorption (2PA), theoretical understanding of the optical parameters enhancement, and practical demonstration of using hydrothermally treated fibrils as a coating material for LED. It opens pathways for elucidating the properties of hydrothermally treated fibril‐based materials, offering innovative solutions that could complement carbon dots in a multitude of applications, thereby advancing the field of sustainable photonics.

Different types of HTP can be distinguished based on their temperature regimes, with the range between 160 and 250 °C referred to as hydrothermal carbonization (HTC).^[^ ^12^ ^]^ In this study, we focused on HTC treatment of β‐lactoglobulin fibrils and monomers, investigating the optical properties of the resulting materials.

In the HTC process, water becomes subcritical, signifying liquid water at temperatures and pressures below its critical point (Tc > 374.15 °C, Pc > 22.1 MPa).^[^ ^13^ ^]^ The pressure exerted by subcritical water must surpass its vapor pressure at a specified temperature to ensure the retention of its liquid phase. In the domain of protein‐solvent dynamics, subcritical water transcends its conventional role as a solvent, concurrently functioning as both a catalyst and a reactant.

At increased temperatures (below 190 °C—above a drastic hydrolysis occurs), proteins typically coagulate into insoluble semi‐soft aggregates. This aggregation reduces the protein surface area exposed to the subcritical water environment, slowing down various degradation processes. Due to their rapid and irreversible coagulation, proteins are significantly more stable in subcritical water compared to room conditions.^[^ ^14^ ^]^

It is well known that various chemical reactions can take place on proteins during HTP with subcritical water. To simplify, only the reactions leading to the formation of cyclic compounds are discussed here, highlighting the potential of hydrothermal treatment (HTT) to enhance optical properties through the creation of larger conjugated systems. For instance, under HTT conditions, glutamine and glutamic acid produce heterocyclic compounds.^[^ ^15^ ^]^ Lysine and arginine yield proline, lactam, or pipecolic acid.^[^ ^16^ ^]^ Aromatic residues exhibit high resistance to HTT; for instance, the imidazole ring of histidine survives even during hydrothermal gasification,^[^ ^17^ ^]^ while phenylalanine can give rise to higher molecular weight compounds.^[^ ^18^ ^]^

Tcherkasskaya^[6a]^ proposed that the emergent optical properties in amyloids can be elucidated by the formation of intrinsic chromophores upon aggregation, with the emissive chromophore being akin to that found in fluorescent proteins such as green fluorescent protein and its analogs. Pinotsi et al.^[6b]^ suggested that fluorescence arises due to the formation of a hydrogen‐bond network, and emission becomes apparent in the visible spectral range upon the development of amyloid fibrils. Conversely, Kumar et al.^[^ ^19^ ^]^ attributed blue‐green fluorescence in proteins to charge recombination, originating even in protein monomers, and involving charged amino acids lysine and glutamic acid. This group also reported a broad, long tail in UV−Vis electronic absorption (250−800 nm), attributed to distance‐dependent charge transfer,^[^ ^20^ ^]^ a phenomenon that became particularly apparent during protein aggregation.^[^ ^21^ ^]^ Finally, Al‐Garawi et al.^[^ ^22^ ^]^ demonstrated that tyrosine can be cross‐linked to a dimer with emission shifted from UV to the blue region of the electromagnetic spectrum.

Additional reactions achievable in a hydrothermal reactor can yield materials with optical parameters better suited for applications in optoelectronics. For instance, the molecules typically used in organic light‐emitting diodes are luminescent organic dyes containing aromatic rings and extended conjugated π‐systems.^[^ ^23^ ^]^ Presently, most of these compounds are derived from petroleum feedstock, and novel emitters based on proteins isolated from industrial side streams, such as β‐lactoglobulin, could present an intriguing alternative as fluorescent protein emitter.^[^ ^24^ ^]^ However, to compete effectively with synthetic molecules in technological applications, the intrinsic optical properties of natural molecules like proteins must undergo significant enhancement.

In this article, we investigated the optical properties of amyloid β‐lactoglobulin fibrils by modifying their internal structure through HTT in a subcritical water environment. We observed that the β‐sheet structural motif typical of amyloids is retained during the HTT of β‐lactoglobulin fibrils, resulting in a unique material not previously described. Furthermore, we explored the structural and optical properties of hydrothermally treated β‐lactoglobulin fibrils **(1)**, comparing them with standard β‐lactoglobulin fibrils **(2)**, as well as with hydrothermally treated native protein monomers **(3)**.

The HTT of samples **(1)** and **(3)** yields easily processable aqueous dispersions of blue emitters. Material **(1)** was then applied as a coating for a commercial LED device to transform UV light into visible light.

By utilizing hydrothermally treated β‐lactoglobulin as an example, we showcased that amyloid protein fibrils, obtained from industrial side streams, could serve as excellent materials that are easily processed for advanced optical applications, paving the way to unexplored scenarios in sustainable organic optoelectronics.

## 2 Results

### 2.1 Hydrothermal Treatment of β‐Lactoglobulin Fibrils

**Figure 1:**
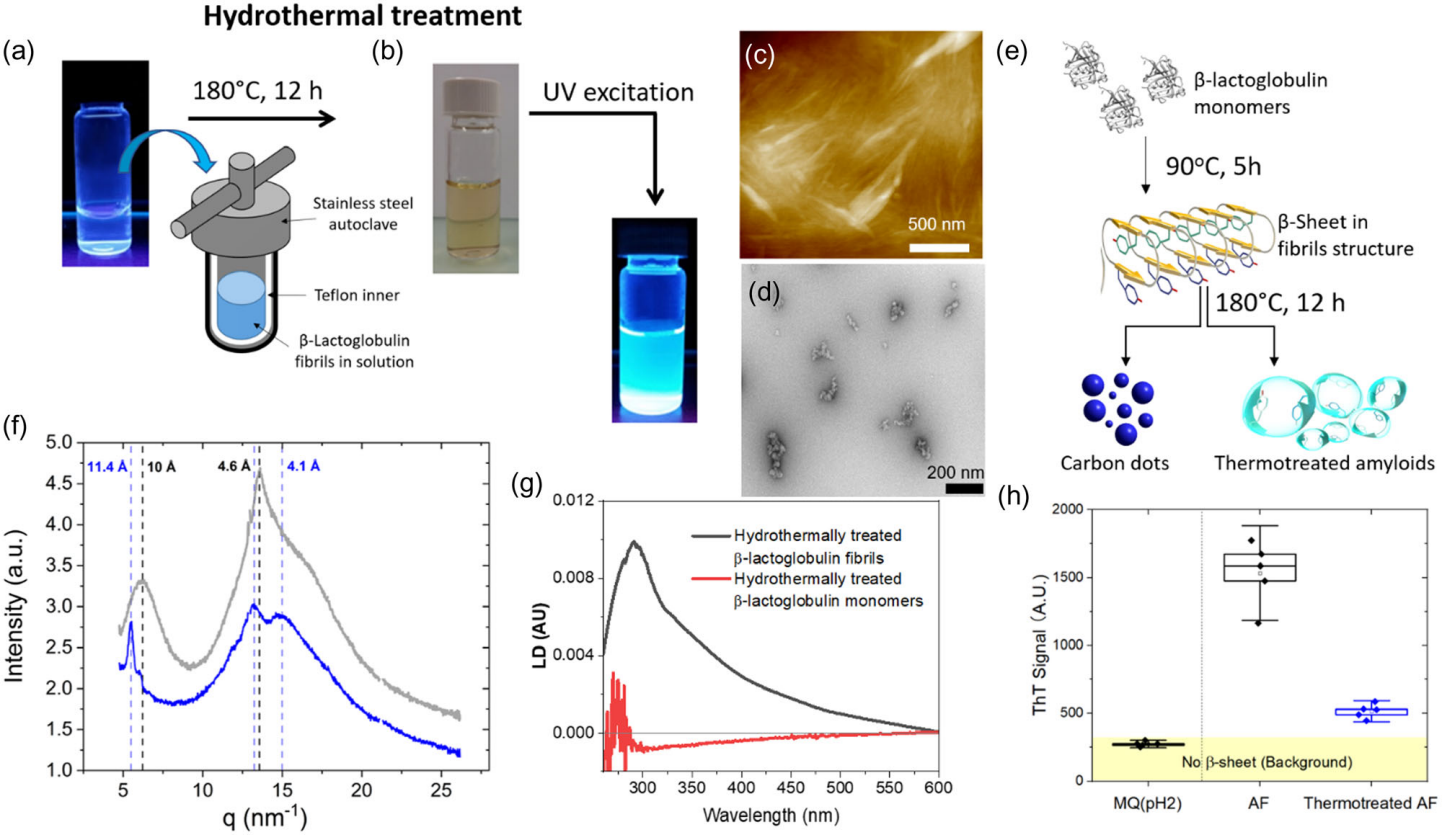
a) Vial with a solution containing β‐lactoglobulin fibrils (**2**) and schematic illustration of the stainless autoclave containing Teflon inner whereby the liquid with fibrils is hydrothermally treated in 180 °C for 12 h, b) the resulting material is a brown solution emitting visible light upon UV irradiation at 366 nm (**1**), c) AFM, d) TEM images of hydrothermally treated β‐lactoglobulin fibrils (**1**), e) schematic illustration of β‐lactoglobulin monomers aggregated into fibrils by heating in 90 °C for 5 h and then transformed by hydrothermal treatment into amyloids and carbon dots, f) wide‐angle X‐ray scattering (WAXS) graphs of β‐lactoglobulin fibrils before (gray) (**2**) and after hydrothermal treatment (blue) (**1**), g) linear dichroism spectra of (**1**) (black) with characteristic absorption peak attributed to aromatic amino acids at 290 nm and LD signal for hydrothermally treated monomers without aromatic amino acid band (**3**) (red), h) ThT fluorescence assay sensitive to β‐sheet content in amyloid fibrils showed enhanced dye emission in presence of both type of aggregates (**1**) and (**2**) at starting protein concentration 0.02 wt%.

The β‐lactoglobulin fibrils were prepared following the standard protocol at low pH (pH 2) and elevated temperature (90 °C, 5 h) **(2)**. Subsequently, the fibrils were transferred to a stainless‐steel autoclave and subjected to HTT at 180 °C for 12 h resulting in a brown color solution which was blue emitting under the UV illumination (**Figure** **1**a,b) **(1)**. These conditions were adjusted in an optimization study whereby different temperatures and reaction time in the autoclave were tested. The objective was to identify the conditions that produce high‐yield amyloid material exhibiting strong blue emission. It was found that 180 °C and 12 h are creating an optimal balance in the autoclave for creating hydrothermally treated β‐lactoglobulin fibrils **(1)**. Subsequently, the monomeric protein was also hydrothermally treated at 180 °C for 12 h **(3)** to serve as a reference for comparison with the fibril material **(1)**.

The high internal pressure in the autoclave allows for the maintenance of the water solution containing the β‐lactoglobulin fibrils in the liquid phase. Figure 1c,d displays the atomic force microscopy (AFM) and transmission electron microscopy (TEM) images of the fibrils after the HTT, respectively. The typical longitudinal fibrillar structure is replaced by smeared and clustered aggregates (Figure 1c). The electron micrographs further indicate that the canonical fibrillar structure is replaced by agglomerates (Figure 1d). Considering these imaging micrographs and the fact that similar hydrothermal conditions are often applied to form carbon dots from organic precursors, a schematic drawing is proposed where amyloids and carbon dots are created simultaneously (Figure 1e).

In Figure 1f, the radially integrated wide‐angle X‐ray scattering (WAXS) curves depict the intensity as a function of the scattering vector (*q*) for hydrothermally treated β‐lactoglobulin fibrils **(1)** and standard β‐lactoglobulin fibrils before treatment **(2)** (crystal images in Figure S1, Supporting Information). The fibrils measured before treatment exhibit the characteristic pattern reported for amyloid fibrils: a peak at 4.6 Å corresponding to the spacing between β‐strands (inter‐strand distance) and another at 10 Å representing the spacing between β‐sheets (inter‐sheet distance).^[^ ^25^ ^]^ Upon HTT of the fibrils, the WAXS pattern undergoes significant changes, although it confirms the retention of the β‐sheet structure. Both the inter‐sheet and inter‐strand distances increased to 11.7 and 4.7 Å, respectively, with the corresponding peaks shifted to lower *q* values. Furthermore, we conjecture that the appearance of a third peak at high *q* (≈15 nm^−1^, corresponding to 4.1 Å) may be associated with weak π–π stacking induced by the thermal treatment.^[^ ^26^ ^]^

In Figure 1g, the linear dichroism (LD) spectra of **(1)** and **(3)** are presented, measured using a flow Couette cell that induces the orientation of elongated molecules in the water flow. LD measures the differential absorption of light polarized parallel and perpendicular to an orientation axis of a sample.^[^ ^27^ ^]^ The LD spectrum, with the maximum corresponding to aromatic amino acids (290 nm), was obtained in the shear flow only for hydrothermally treated fibrils **(1)**, while in **(3)**, there was no signal in the aromatics band. LD indicates that the fibril structure is retained, with aromatic residues being oriented parallel to the fibril axis, whereas HTT of monomer β‐lactoglobulin leads to complete decomposition to carbon dots (Figure 1g).

The fluorescence enhancement of ThT in the presence of **(1)** confirms the presence of β‐sheet structure leading to enhanced fluorescence signal, as expected for amyloids (Figure 1h and S2, Supporting Information). However, in Fourier‐transform infrared spectroscopy measurements (Figure S3, Supporting Information) lack of the characteristic amide I peak at 1611–1630 cm^−1^ for the sample after HTT suggest that the treatment itself alter—and possibly partially decompose—most of the β‐sheets, rendering them undetectable to this less sensitive spectroscopic technique. Circular dichroism (CD) reflects that random coils are predominant (Figure S3, Supporting Information).

### 2.2 Enhancement of the Optical Properties

**Figure 2:**
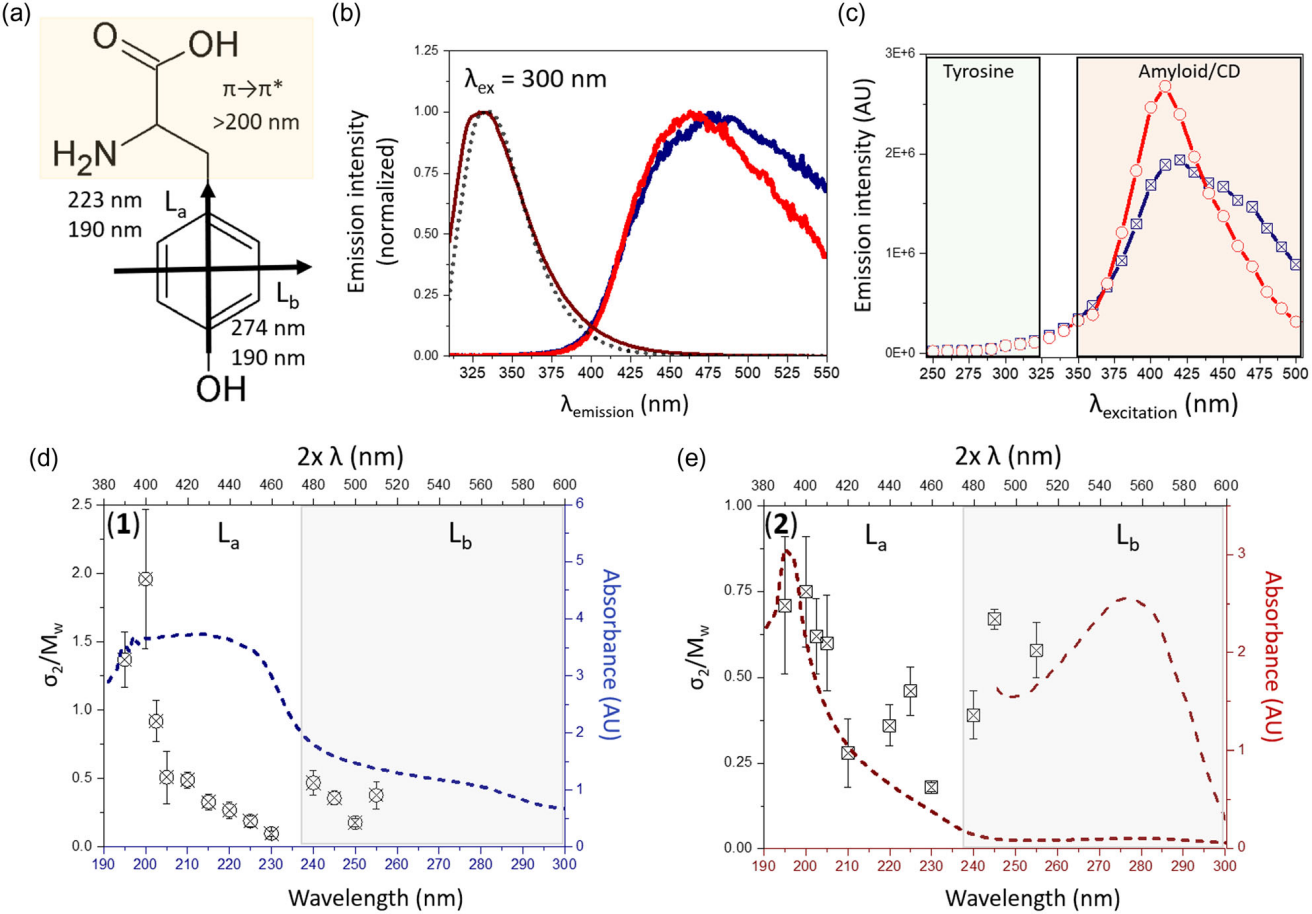
a) The structure of tyrosine amino acid with the marked electronic transitions of the phenol ring of the tyrosine (black arrows) and π → π* transitions of the peptide backbone (marked in yellow), b) fluorescence spectra of the hydrothermally treated β‐lactoglobulin fibrils (**1**) (dark blue), standard β‐lactoglobulin fibrils (**2**) (brown), native protein monomers (dotted black) and hydrothermally treated β‐lactoglobulin monomers (red) (**3**). The excitation wavelength was 300 nm, c) maximum fluorescence intensity measured at different excitation wavelengths in the range of 250 to 500 nm for (**1**) (dark blue crossed squares) and (**3**) (red open dots), d) two‐photon absorption (2PA) spectra of hydrothermally treated β‐lactoglobulin fibrils (**1**), and e) standard β‐lactoglobulin fibrils (**2**). 2PA was measured in the absorption range between 390 and 510 nm, together with the one‐photon spectrum (dashed lines) replotted from the normal absorption spectrum with the wavelength multiplied by a factor of two and normalized to the peak height of 2PA for comparison. The tyrosine absorption band in e) is magnified in region 240–300 nm. The bottom *x* axis corresponds to one‐photon absorption wavelength range and top *x* axis corresponds to two‐photon absorption wavelength range.

The structural characterization indicates that the internal morphology of aggregates was reorganized as a consequence of the HTT. In the next step, optical properties were examined. **Figure** **2** summarizes the results on intrinsic fluorescence and 2PA.

The intrinsic fluorescence was measured in hydrothermally treated β‐lactoglobulin fibrils **(1)** and compared with hydrothermally treated native protein monomers **(3)**, as well as with standard β‐lactoglobulin fibrils **(2)** and monomers. The emission spectra were measured with excitation wavelengths ranging from 230 to 500 nm, revealing three emissive species.

When the samples are excited between 230 and 320 nm, the emission arises from the absorption band of aromatic amino acids. This band consists of two characteristic transition dipole moments, *L*_b_ and *L*_a_ (Figure 2a).^[^ ^28^ ^]^ The emission excited above 320 nm, not attributed to aromatic amino acids, is often referred to as intrinsic fluorescence. The origin of this fluorescence is still debated, and recent findings are summarized in the introduction section. In the case of hydrothermally treated proteins, the interpretation of intrinsic fluorescence is particularly challenging because the emission can result from protein aggregation and carbonization. The latter leads to the formation of carbon dots that emit blue‐green light.^[^ ^29^ ^]^

The UV‐excited fluorescence spectra in each β‐lactoglobulin form are dominated by aromatic residues (Figure 2b). A typical emission band of aromatic chromophores, primarily tyrosine‐dominated, was observed in β‐lactoglobulin monomers and fibrils **(2)**. The emission was 120 nm red‐shifted in hydrothermally treated samples **(1)** and **(3)**. According to the literature, the fluorescence spectral shifts are attributed to the formation of covalently cross‐linked tyrosines created under hydrolytic conditions.^[^ ^30^ ^]^

Figure 2c illustrates that the maximum emission in **(1)** and **(3)** was achieved by exciting samples at 410–420 nm. These excitation wavelengths are commonly reported in studies on amyloid intrinsic fluorescence,^[7b]^ as well as in investigations on the emission properties of carbon dots.^[^ ^28^ ^]^ In cases where amyloids and carbon dots are in a mixture, there is spectral overlapping of the fluorescence between the two materials, making the differentiation of thermally treated fibrils from carbon dots impossible. This is particularly challenging since carbon dots have a significantly higher quantum yield than amyloids, likely dominating the fluorescence in hydrothermally treated samples (Figure S4, Supporting Information).^[^ ^31^ ^]^

Nevertheless, differences in fluorescence properties between **(1)** and **(3)** when excited beyond 360 nm indicate that the structural character of the starting material is crucial for obtaining unique optical properties in the HTT process. In fact, the results suggest that the precursor material is modified, but the major optical characteristics of amyloid fibrils are preserved. A typical spectral tail attributed to the protein aggregates causing light scattering was detected in the emission of **(1)** but not in **(3)** (Figure S5a, Supporting Information). Spectral shifts and lifetimes measured in the tyrosine band, which are sensitive to the local environment and polarity, indicate that sample **(1)** is very different in its nature when compared with **(3)** in terms of photophysics (Figure S5b, Supporting Information).

Following the optical characterization of hydrothermally treated β‐lactoglobulin fibrils, the 2PA cross section was determined using a combination of the closed‐ and open‐aperture *z*‐scan method.^[^ ^30^ ^]^ The *z*‐scan method is a standard technique to determine the nonlinear absorption coefficient (σ_2_). The unit for the 2PA cross section is named the GM, derived from the name of the contributor in the field of nonlinear optics, Maria Goeppert Mayer. One GM is σ_2_ = 10^−50^ cm^4^ s photon^−1^ (for details about *z*‐scan and schematic illustration of the experimental setup see the SI and scheme S1, Supporting Information).

First, the *z*‐scan was calibrated on a fused silica plate (2 mm thick) and a quartz cell filled with the solvent alone: pH = 2 water buffer, which was later used for dissolving the β‐lactoglobulin. This step is necessary to adjust the energy in the focal point of the *z*‐scan measurement to avoid nonlinear absorption arising from glass or solvent alone. Next, the *z*‐scan was measured in β‐lactoglobulin fibrils before **(2)** and after HTT **(1)**. As a control, the native monomers and hydrothermally treated monomers **(3)** were also examined. The measurements were performed every 5 nm in the spectral range of 390–510 nm to draw a 2PA spectrum (Figure 2d,e). The first *z*‐scan was performed at 510 nm (Figure S6, Supporting Information). Sample **(1)** and **(2)** show 2PA, and no signal was detected in monomeric samples (data not shown). The 2PA σ_2_ cross sections were scaled over the molecular weight (*M*_w_) of the β‐lactoglobulin monomer for standardization.^[^ ^32^ ^]^ In the case of untreated fibrils **(2)**, the σ_2_/*M*_w_ monomer scaled value was 0.58, and for the hydrothermally treated fibrils **(1),** it was 0.38. Scaled values were comparable to previously reported in fibrils (insulin, lysozyme, and α‐synuclein) at 510 nm.^[5a]^

Subsequently, the *z*‐scans were measured in the absorption band below 500 nm, where the *L*_a_ transition is the second low‐lying transition moment of the aromatic tyrosine residue (Figure 2a). In contrast to the standard fibrils **(2)**, the hydrothermally treated fibrils **(1)** exhibited strong 2PA wavelength dependency in the absorption band associated with the *L*_a_ transition. Starting from longer wavelengths in the *L*_a_ range of 380–470 nm, the 2PA constantly rose, reaching its maximum at 400 nm where scaling was σ_2_/*M*_w_ = 1.96. Close to 400 nm, additional transitions of the phenol ring, *B*_a_ and *B*_b_, as well as π→π* transitions of the peptide backbone, can influence the 2PA (Figure 2a). The results are interpreted in terms of transition dipole moments of the aromatic residue—tyrosine and its covalently cross‐linked multimers (di‐tyrosine, tri‐tyrosine, etc.). These findings will be collectively discussed in the following sections alongside fluorescence results and quantum chemical calculations on 2PA in tyrosines.

### 2.3 Quantum Chemical Calculations on 2PA Enhancement Mechanism

While optical phenomena in amyloids are extensively documented, a comprehensive explanation for the unusual properties of fibrils remains elusive. The complexity of the system at the molecular level, coupled with a general lack of structural information about the internal organization of chromophores in the fibrils, makes it challenging to deduce the underlying mechanisms responsible for amyloid photonics. In an effort to illuminate potential mechanisms for nonlinear absorption enhancement, quantum chemical calculations were employed here.

Calculations were conducted using a dimer tyrosine model since the software is capable only of simulating interactions between small chromophores in the context of nonlinear absorption. To ensure relevance to the molecular organization of aromatic chromophores in amyloid fibrils, the dimer system for calculations was isolated from crystallographic data obtained for aggregated peptide. It is important to note that the calculations consider only two amino acid residues and their displacement in space, offering insight into some influence on nonlinear absorption. However, although this allows to make the problem tractable, it falls short of fully explaining the overall photonic parameters of amyloids.

**Figure 3:**
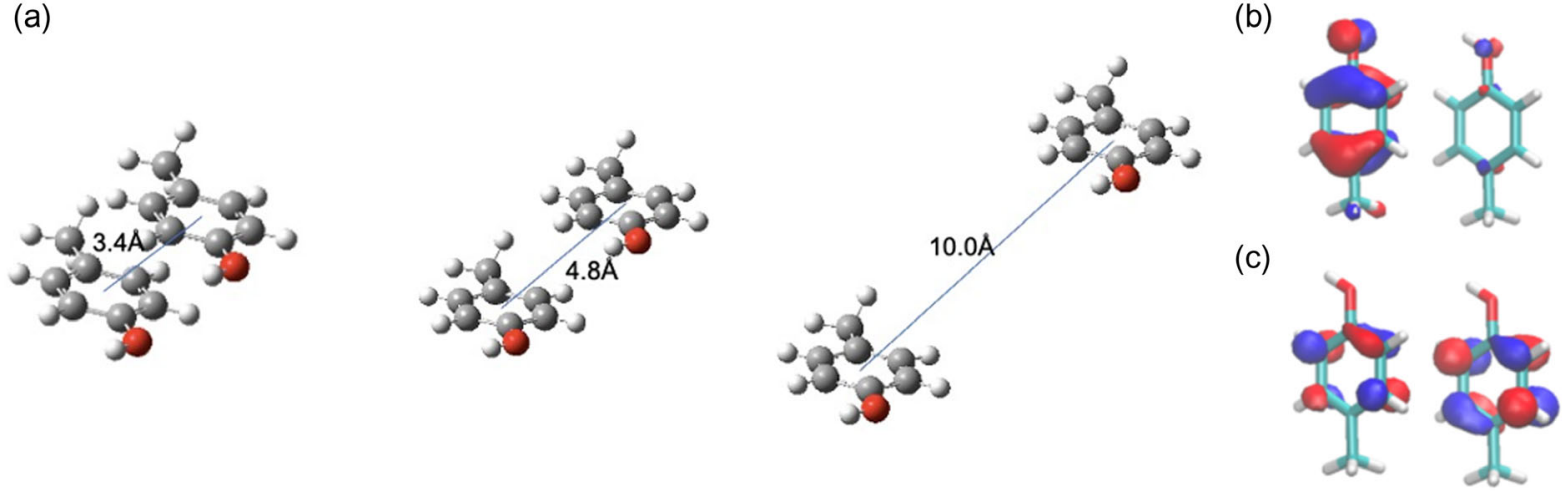
a) The molecular structure of the tyrosine dimer at different interspace distances between two molecules. The distances between the centers of the phenol rings are 3.4, 4.8, and 10 Å in the geometry shown, b) HOMO‐1, and c) LUMO + 1 states for the dimer at 4.8 Å. Images were made with VMD using a contour value of 0.05 for the orbital plots.

Dimers were constructed by duplicating the monomer and translating the coordinates of the duplicate by 3.4, 4.8, and 10 Å in the *y*‐direction from the crystal structure coordinates.^[^ ^33^ ^]^ Translating 4.8 Å in the *y*‐direction corresponds to the actual distance and orientation of two adjacent tyrosine residues in the crystal structure of the β‐sheets aggregates. Molecular structures of the tyrosine dimer at different distances are shown in **Figure** **3**a.

Before delving into the discussion of 2PA in the dimer at various intermolecular distances, it is convenient to examine the nature of orbitals. The highest occupied molecular orbital (HOMO) is a π‐orbital located predominantly on one of the tyrosine rings, while the HOMO‐1 is a π‐orbital located predominantly on the other tyrosine ring. The HOMO and HOMO‐1 are nearly degenerate. The lowest unoccupied molecular orbital (LUMO) and the LUMO + 1 are π*‐orbitals delocalized over the two tyrosine rings, with (almost) equal density on each. The first excitation in the dimer at 4.8 Å is primarily a HOMO → LUMO excitation. Intermolecular charge transfer is evident in density functional theory calculations on the dimer. The ICT excitations with the largest 2PA strength are from the HOMO and HOMO‐1 into the LUMO + 1, with the highest 2PA strength being an excitation from the HOMO‐1 (located on one monomer) to the LUMO + 1 (delocalized over both monomers). The HOMO‐1 and LUMO + 1 from time‐dependent density functional theory calculations are shown in Figure 3b,c, respectively.

The tyrosine dimer exhibited the strongest 2PA excitation peaks between 370 and 405 nm, depending on the interspace between adjacent chromophores.

In the case of the first 15 excitations at a 10 Å interspace, the highest 2PA strength was at 403 nm, with a σ_2_ of 27 GM. This result suggests a weak intermolecular charge transfer between tyrosine molecules when they are separated by a distance of 10 Å, and there is no significant communication between the aromatic amino acids in the context of 2PA enhancement.

At 4.8 Å interspace, the highest 2PA occurred at 370 nm, reaching 218 GM, and it was one order of magnitude larger than at 10 Å intermolecular distances. Further shortening the distance to 3.4 Å led to a substantial enhancement of 2PA, with σ_2_ = 2630 GM at 380 nm. The orbital analysis indicates that these excitations are intermolecular charge‐transfer peaks from one tyrosine ring to the other. Extending the model system to three tyrosines led to further enhancements in 2PA values in the same wavelength region as found for the dimer, confirming the general concept of distance‐dependent cooperative enhancement of nonlinear absorption by aromatic residues.

### 2.4 Hydrothermally Treated Fibrils in LED Optoelectronics

In addition to modifying optical characteristics, HTT also alters the material properties of proteins. The synergy between optical attributes and material properties is beneficial for the fabrication of LED coatings. One potential method for the fabrication of LED coatings is to develop gels that can be applied directly onto the LED.

To examine the effects of HTT on material properties, specifically on macro‐ and microrheological behavior, dynamic shear rheology and multispeckle diffusing‐wave spectroscopy (MS‐DWS) techniques were employed. Shear rheology revealed the highest viscosity in untreated fibrils **(2)**. Thermal treatment resulted in a decrease in viscosity, indicative of material degradation (Figure S8, Supporting Information). Rheolaser MASTER^TM^, leveraging MS‐DWS technology, highlighted notable distinctions between fibrils and monomers subjected to hydrothermal treatment (for details se SI and Figure S9, Supporting Information).

**Figure 4:**
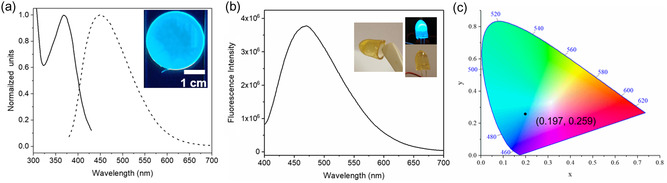
a) Normalized excitation and emission spectra of film made of (**1**):PVA:GLY in a ratio of 1:4.5:38, solid line is the excitation spectrum (emission at 448 nm), and dashed line is the emission spectrum (excitation at 365 nm). The inset shows a photographs of a freestanding film under UV‐light, b) fluorescence spectrum of a coating excited by a UV‐LED (365 nm) operated at 3.5 V. The inset shows photographs of a freestanding gel, as well as the same gel attached to a UV‐LED with lights on (right top) and off (right bottom). c) The corresponding CIE diagram (CIE 1931) of the LED devices operation under 3.5 V.

The HTT of β‐lactoglobulin fibrils results in a material that, upon UV excitation, exhibits enhanced emission in the visible part of the spectrum. This indicates that hydrothermally treated fibrils can be employed for applications related to the conversion of UV light to visible light, such as coatings on ultraviolet light‐emitting diodes (UV‐LEDs). Since the material **(1)** is obtained as an aqueous dispersion, a convenient method for preparing gels that can be cast into various shapes is to mix **(1)**, dispersed in water, with an aqueous solution of polyvinyl alcohol (PVA, *M*_w_ = 115 000) and glycerol (GLY). The resulting soft material can then be cast into films (by casting in, for example, a Petri dish) or an LED coating (by casting in a suitable mold, e.g., an Eppendorf tube). The concentration of **(1)** in the coating films can be modified by mixing **(1)** with PVA/glycerol in different ratios. The optimal material combination for LED coatings was found to be a mixture of **(1)**: PVA:GLY in a ratio of 1:4.5:38. In **Figure** **4**a, photoluminescence spectra of a freestanding film are displayed, while in Figure 4b, the spectrum of the coating material applied on a commercial UV‐LED (365 nm) is shown. In the inset are photos of the freestanding LED coating and the coating applied onto an LED. Figure 4c presents the CIE coordinates (0.197, 0.250) of the LED device operated at 3.5 V.

The results on coatings and films indicate that the material can be easily processed from a solution to obtain a functional layer for converting UV light of commercial LED into visible blue emission. By adjusting the concentration of **(1)** and using appropriate mixing ratios with PVA and glycerol, the gel film can be utilized to tune the optical properties of commercial LEDs. At relatively low concentrations of **(1)**, much of the UV light from the LED was not absorbed. On the other hand, an excessively high concentration of **(1)** led to the quenching of visible luminescence. It should be noted that the LED coatings have a thickness in the millimeter range, and the UV light emitted from the LED passes through the coating. This means that light can be absorbed, emitted, reabsorbed, and reemitted by **(1)**, resulting in unique properties that can be used for optical tuning.

## 3 Discussion

Assembling proteins into amyloid fibrils creates a network of hydrogen bonds and stacking of aromatic residues, resulting in remarkable mechanical properties with Young's moduli values in the order of several GPa.^[^ ^34^ ^]^ The tensile strength of individual fibrils is comparable with that of steel, and their mechanical shear stiffness is similar to that of silk.^[^ ^35^ ^]^ The amyloid fibril is a relatively stable structure held together by multiple hydrogen bonds, hydrophobic interactions, and other types of interactions. The role of hydrogen bonds and stacking in the secondary structure of biomolecules can be illustrated using DNA as an example. The melting temperature of DNA is directly correlated to the number of nucleobases in the DNA chain, where a longer chain implies stronger bonding that improves duplex stability.^[^ ^36^ ^]^ A somewhat similar reasoning can be applied to amyloid fibrils in the hydrothermal process, where entropy driving forces tend to disassemble fibrils, but hydrogen bonds and π‐stacking counteract complete degradation. The exact geometry of the fibril's internal structure after exposure to hydrothermal conditions is the result of intra‐ and intermolecular interaction vs entropic dissociative contributions.^[^ ^37^ ^]^

The morphology in hydrothermally treated fibrils was revealed by WAXS, showing inter‐sheet and inter‐strand distances of 11.7 and 4.7 Å, respectively, with a third peak at 4.1 Å. Compared to normal amyloid fibrils, the inter‐strand distance increased by roughly 1.7 Å, and the inter‐strand distance increased by 0.1 Å. The presence of a third peak at 4.1 Å could indicate that a relatively weak type of π‐stacking interaction in a large number of stacked aromatic residues multiplies the effect, creating a strong stabilizing force that helps to preserve the β‐sheet structure in autoclave conditions.^[^ ^38^ ^]^ However, it should be noted that as the fibrillated material also contains peptide fragments, this is a highly complex multicomponent system. Additionally, dimerization, oligomerization, cyclodimerization, and cyclooligomerization are commonly observed reactions in autoclave conditions. Although highly speculative, Korner^[^ ^15^ ^]^ suggested that the incorporation of decomposition products in the HTT process is conceivable, and some residual amino or carboxy groups could be involved in oligocondensation reactions. Any condensation or cyclization will certainly lead to the modification of the optical properties of amyloid fibrils. However, due to the structural complexity of protein aggregates, staying with such an explanation might not be fully satisfying. The presence of multiple chromophores and the ability of biopolymers to self‐assemble into various structures contribute to the elusive nature of the physical origin of many optical properties of biomolecules.

From the state‐of‐the‐art knowledge, it is well known that aromatic amino acids exhibit fluorescence, as well as 2PA.^[^ ^39^ ^]^ Extensive theoretical and experimental studies have shown that interactions between aromatic residues play a central role in the assembled state, influencing their optical properties.^[^ ^40^ ^]^ There is a growing number of articles reporting intrinsic fluorescence or nonlinear absorption in natural molecules,^[^ ^41^ ^]^ including various forms of proteins^[^ ^42^ ^]^ and peptides,^[^ ^43^ ^]^ as well as in their building blocks—amino acids.^[^ ^44^ ^]^ Several hypotheses have been suggested to explain the origin of the emergent optical properties of amyloids. Recalling them: 1) formation of intrinsic chromophores upon aggregation^[6a]^; 2) cross‐linking to a tyrosine dimer in harsh external conditions^[^ ^30^ ^]^; 3) creation of the hydrogen‐bond network upon formation of amyloid fibrils^[6b]^; and 4) through‐space cooperative effects of aromatic residues, mostly exciton coupling of tyrosines.^[5a]^

In native fibrils, the scenario with through‐bond enhancement was dismissed due to the large distance between the tyrosines in the protein sequence and the negligible influence of π‐conjugation on 2PA. However, in light of the presented results on hydrothermally treated fibrils, the impact of π‐conjugation on the optical properties of fibrils needs to be reconsidered.

The assembly of tyrosines and their through‐bond and through‐space interactions is discussed to elucidate the enhancement of optical properties in hydrothermally treated amyloid fibrils. It is noteworthy that, among all amino acids, tyrosine exhibits the highest resistance to hydrothermal decomposition.^[^ ^45^ ^]^ This resilience is particularly significant in the context of fibril restructuring under autoclave conditions.

The fluorescence experiments conducted on the hydrothermally treated samples did not exhibit the typical emission band associated with tyrosine monomers. Instead, a band with maximum fluorescence at approximately 470 nm was observed,^[^ ^46^ ^]^ indicating the presence of cross‐linked multimers of tyrosine (Figure 2b). These multimers, up to pentamers, have been reported previously. Such cross‐links are known to efficiently stabilize the β‐sheet structure in fibrils, making them resistant to catalytic reaction.^[^ ^47^ ^]^ The π‐conjugated tyrosines observed here resemble the extended π‐electron systems reported in organic dendrimeric structures, where a strong enhancement of 2PA was observed upon increasing the number of aromatic rings through chemical bonding.^[^ ^48^ ^]^ Quantum calculations on the tyrosine dimer suggest that a parallel displacement geometry is the most efficient configuration for enhancing nonlinear absorption. The third peak at 4.1 Å in WAXS and the fluorescence experiments both point to a reduced interspace distance in the π‐stacked system. In standard fibrils or peptide dimers, the inter‐strand distance is typically between 4.6 and 4.8 Å. The shorter through‐space distance between aromatic residues promotes a significant increase in nonlinear absorption, as revealed by *z*‐scans and confirmed by quantum chemical calculations. In the hydrothermally treated sample, the 2PA rises significantly below 470 nm, corresponding to the *L*_a_ transition of the aromatic residue. The highest σ_2_ value was detected in the spectral region of 390–400 nm, suggesting that the *L*_a_ transition has a certain contribution from *B*_a_ and *B*_b_ transitions, lying just below 200 nm in the linear spectrum. The directions of *B*_a_ and *B*_b_ transitions are both perpendicular and parallel to the symmetry axis of the phenol ring. Additional absorption peak can appear in the *L*_a_ band, borrowing intensity from the *B*_a_ and *B*_b_ transitions.^[^ ^49^ ^]^ Multiple of such transitions can be 2PA allowed, significantly boosting 2PA. Moreover, it was found that *B*_a_ and *B*_b_ of the phenyl ring can undergo shifts in bulk solvent to the blue and to the red, respectively.^[^ ^50^ ^]^ Thus, one can expect strong 2PA enhancements coming from *B*_a_ and *B*_b_ and its contribution to the *L*_a_ absorption band near 400 nm due to solvent effects and different microenvironments around aromatic units in amyloids structure.

## 4 Conclusion

β‐Lactoglobulin fibrils were subjected to HTT in a subcritical water environment, resulting in the transformation of fibrils into partially disassembled fibrils. The hydrothermally treated fibrils **(1)** exhibited blue‐green fluorescence and enhanced 2PA. These optical effects were attributed to the cross‐linking of tyrosines, leading to cooperative effects through both through‐bond and through‐space interactions. The 2PA was primarily related to intermolecular charge transfer of tyrosine units, and quantum calculations revealed that the enhancement in 2PA was strongly correlated with the interspace distance between aromatic residues.

A key feature to allow the use of biomolecules in photonics is to enhance their optical properties: that was achieved in the hydrothermal process. Hydrothermally treated composite material was used as a coating for a commercial LED device and UV irradiation was successfully converted to blue‐green emission of light.

The majority of applied phosphors are inorganic materials, often rare earth metals with many of these being classified as critical materials.^[^ ^51^ ^]^ There is accordingly a large interest in developing phosphors based on readily available bio‐based materials.^[^ ^52^ ^]^ Herein, HTP was presented as a cheap and simple method to turn amyloids into materials readily applicable in photonics.

In photonics, the most attractive option would be to employ materials available in large quantities as industrial side or waste streams.^[^ ^53^ ^]^ The use of readily available bio‐based materials, such as β‐lactoglobulin derived from whey (a side‐stream from the dairy industry), is particularly promising. The HTT at relatively mild temperatures enhances the photophysical properties of the composite material, showcasing the potential for sustainable and large‐scale production of bio‐based materials for photonics applications.

## Conflict of Interest

The authors declare no conflict of interest.

## Supporting information

Supplementary Material

## Data Availability

The data that support the findings of this study are available from the corresponding author upon reasonable request.
