# Supplementary material for: Photonics of Hydrothermally Treated β‐Lactoglobulin Amyloids
Source: Small Sci. 2024 Apr 24;4(7):2400054. doi: 10.1002/smsc.202400054 (PMC11935170; doi:10.1002/smsc.202400054)
Supplement: Supplementary file 1 — Supplementary Material [file SMSC-4-2400054-s001.pdf]

Photonics of Hydrothermally Treated β-lactoglobulin Amyloids

Piotr Hanczyc[1,6*], Serena R. Alfarano[2], Sreenath Bolisetty[2] Jiangtao Zhou[2], Mohammed Peydayesh[2], Viviane Lutz-Bueno[3], Ana Diaz[3], S. Roy Goswami[2], Maarten. T. P. Beerepoot[4], Mehboob M. Alam[4,5], Lei Wang[6], Niclas Solin[6], Iwona Szymanska[7], Raffaele Mezzenga[2,8*]

[1] Institute of Experimental Physics, Faculty of Physics, University of Warsaw, Pasteura 5, 02-093 Warsaw, Poland

[2] ETH Zurich, Department of Health Sciences and Technology, Zurich, Switzerland

[3] Paul Scherrer Institut, Villigen PSI, Switzerland

[4] Centre for Theoretical and Computational Chemistry, Department of Chemistry, University of Tromsø – The Arctic University of Norway, N-9037 Tromsø, Norway

[5] Department of Chemistry, Indian Institute of Technology Bhilai, Durg, Chhattisgarh 491001, India

[6] Department of Physics, Chemistry, and Biology, Electronic and Photonic Materials, Biomolecular and Organic Electronics, Linköping University, Linköping, 581 83, Sweden

[7] Department of Food Technology and Assessment, Institute of Food Sciences, Warsaw University of Life Sciences (WULS-SGGW), 159C Nowoursynowska St., 02-776 Warsaw, Poland

[8] ETH Zurich, Department of Materials, Zurich, Switzerland

Materials and methods

β-lactoglobulin (β-lg, purity ≥90%) was extracted and purified from whey protein (Fonterra, New Zealand) according to a previously developed protocol[1]. β-lactoglobulin fibrils were prepared by dissolving 2% weight of the monomer protein in pH2 HCl buffer. The protein was then incubated in 90°C for 5 h, until the viscous solution was obtained. In the next step the β-lactoglobulin fibrils were transferred to the stainless-steel autoclave and treated with high temperature of 180 °C for 12 h.

Atomic Force Microscopy: Freshly cleaved mica was functionalized by an aliquot (10μl) of 0.1% (3-Aminopropyl) triethoxysilane (APTES) for 2 min, rinsed with Milli-Q water (pH 2) and dried by a gentle air flow. Immediately, another aliquot (10μL) of hydrothermal treated amyloid fibril solution at the concentration of 1 wt% was deposited on mica for 2 min, rinsed with Milli-Q water and dried by a gentle air flow.

AFM measurements were performed by a Bruker multimode 8 scanning probe microscope (Bruker,USA). AFM imaging was operated in soft tapping mode under the ambient condition, using a commercial silicon nitride cantilever (Bruker, USA) at a vibration frequency of 150 kHz, and a relatively soft tip-sample interaction was applied. AFM images were flattened using Nanoscope 8.1 software (Bruker, USA), and no further image processing was applied.

Thioflavin T Assay: A total volume of 100μL solution at different concentrations with 20 μM ThT was transferred to 96-well microplate plate (Corning 96-wellplate, Corning Inc.). ThT signal of the solution was monitored in a ClarioStar plate reader (BMG Labtech, DE) by recording ThT fluorescence emission at 480 nm with excitation at 440 nm.

Circular Dichroism Spectroscopy: CD spectroscopy was carried out with a Jasco J-815 CD spectrometer in a range of 190–280 nm, at a resolution of 0.2 nm in the continuous scanning mode. An aliquot of thermal treated amyloid solution at the concentration of 0.01 wt% was investigated in a quartz cuvette with an optical path length of 1 mm. Four spectra were averaged to obtain the final spectrum.

Fourier-transform infrared spectroscopy (FTIR): The FTIR measurements were performed using a Varian 640 suitable for FTIR spectroscopy. The spectra were acquired in attenuated total reflection (ATR) geometry using a diamond crystal as ATR unit. Spectra were collected from 1000 cm^{-1} to 4000 cm^{-1} with an average of 128 scans and a resolution of 4 cm^{-1}. The background was measured and subtracted automatically. Measurements were performed at ambient conditions.

Absorption spectra: were recorded on a JASCO spectrophotometer.

Steady-state fluorescence: spectra were recorded in custom-built experimental setup. For recording fluorescence spectra samples were excited with light of a xenon arc lamp passing through a monochromator with the central wavelength and transmission bandwidth set to 405 nm and 16 nm, respectively, and a short-pass absorption filter (absorption edge at 450 nm). The emitted light was analysed with a SpectraPro 150 Czerny-Turner imaging monochromator equipped with a CCD camera (Andor DU420A-BU2). The fluorescence light was collected at the right angle to the direction of the excitation light and the studied thin film was oriented at 45° angle with respect to the excitation and detection directions. A long-pass filter (edge at 430 nm) in front of the monochromator was used to eliminate scattered excitation light.

Time-resolved fluorescence: Lifetime decays were recorded using a custom-built setup based on the PicoQuant HydraHarp 400 Multichannel Picosecond Event Timer and the picosecond diode laser PicoQuant LDH-P-C-405B working at 405 nm. Repetition frequency of the laser was set to 10 MHz. Thin film samples were oriented at approx. 45° with respect to the excitation beam, with the fluorescence light collected at the right angle to the direction of excitation. The collected light passed through a long-pass cut-off filter in order to remove scattered excitation light. The setup was also used for measurements of liquid samples, therefore the fluorescence light passed through a polarizer, whose transmission axis was oriented at the magic angle (54.7^o) with respect to the polarization of the excitation beam, in order to eliminate artifacts due to rotational diffusion. The fluorescence light was focused on the entrance slit of a Czerny-Turner monochromator (Acton Research SpectraPro 150) used to select a given wavelength from the fluorescence spectrum. After the monochromator the light was detected with the Hamamatsu R3809U-50 photomultiplier sensitive in the 160-850 nm range. The output signal from the photomultiplier was amplified with the Ortec Model 9327 amplifier prior to routing it to the HydraHarp module. The overall instrumental response function, measured by scattering the excitation light in a suspension of titanium dioxide, was narrower than 100 ps FWHM.

Z-scan: The stock solutions of β-lactoglobulin fibrils were used for the nonlinear experiments. Nonlinear absorption experiments were carried out using a laser system consisting of a femtosecond pulses generated by an amplified femtosecond system (Coherent Legend Elite Duo) at 5 kHz repetition rate, approx. 50 fs long (FWHM) operating as an 800 nm pump and Opera SOLO crystal-based optical parametric amplifier. Samples were placed in 1 mm path length Starna quartz glass cuvettes, stoppered, and sealed with Teflon tape. Results obtained on the cells with β-lactoglobulin were calibrated against Z-scan measurements performed on a fused silica plate (2 mm thick) and compared with the measurements on an identical glass cell filled with the solvents alone: pH=2 water buffer. The output from the Opera Solo

in the range from 400 nm to 520 nm was appropriately filtered using wavelength separators and colour glass filters to remove unwanted wavelength components, attenuated to µJ/pulse range and used as excitation source for simultaneous recording of standard open-aperture (OA) and closed-aperture (CA) Z-scan traces.

Before entering the setup, the beam passes through a telescope (made from lens with focal lengths of 100 mm and 200 mm, respectively) (not visible in the diagram), which enlarges the beam twofold. This reduces the beam waist in the next part of the setup. Additional neutral density filter allows regulation of the beam intensity. The beam passes through a beam splitter (BS, first on the left), which redirects a part of it (approx. 4%) onto a reference photodiode (PD, first on the left). The signal from the photodiode is used to reduce signal noise caused by the beam power fluctuations. Other part of the beam id directed on a lens (focal length 125 mm) (S, lower part of the diagram), after which a beam waist is formed. The waist is utilised to measure the properties of a sample (P). Under the beam a translation stage (movement range 200 mm), which allows changing the sample position in respect to the beam waist. After passing through the material, the beam is partially redirected (BS, on the right bottom) to a circular aperture (I). The aperture allows only the central part of the beam to pass ("closed aperture" arm). After the aperture the beam is directed onto a photodiode (PD). The other half of the beam is directly passed onto the another photodiode (PD, first on the right) ("closed aperture" arm). In front of each photodiode a lens (each with focal length of 125 mm) (S) is placed which focuses the beam onto the photosensitive area of the photodiode. Each photodiode utilises also a neutral density filter, which prevents saturation of a photodiode. During the measurements diodes of a type S2386 were used, with spectral range from 320 nm to 1100 nm. After loading them with resistors (100 kΩ), output pulse amplitude was 0.6 V to 2.5 V. The output was kept (by the means of neutral density filters) in this range when the input beam power was changed. Measurement was governed by a program written in the LabVIEW software. The program was driving an ADC card which was triggered by the synchronisation pulses from the laser. The sample movement was also done automatically. Since the beam exhibited some movement caused by the convection of air, and these fluctuations significantly altered the signal recorded in the CA, the program was first, making a series of measurements to establish a mean value of the signal, and then, only those values were recorded, which were not laying too far from the mean value. Data points were collected and averaged for consecutive scans. Correction for absorption influence on the nonlinear refraction index coefficient measurement was done by dividing the signal from CA by the signal from OA.

The beam was focused so as to provide a focal spot in the range w_0 25 - 50 µm (giving the Rayleigh range which was always taken well in excess of the total thickness of the cell or the reference silica plate) and the cuvette was made to travel in the Z- direction, typically from -10 to 10 mm. The outputs were fed into three channels of a digital oscilloscope and the data were collected by a computer using custom LabVIEW software. The traces of the CA and OA scans obtained by dividing each of them by the laser input reference were analyzed with the help of a custom fitting program that used equations derived by Sheik-Bahae et al.[2]

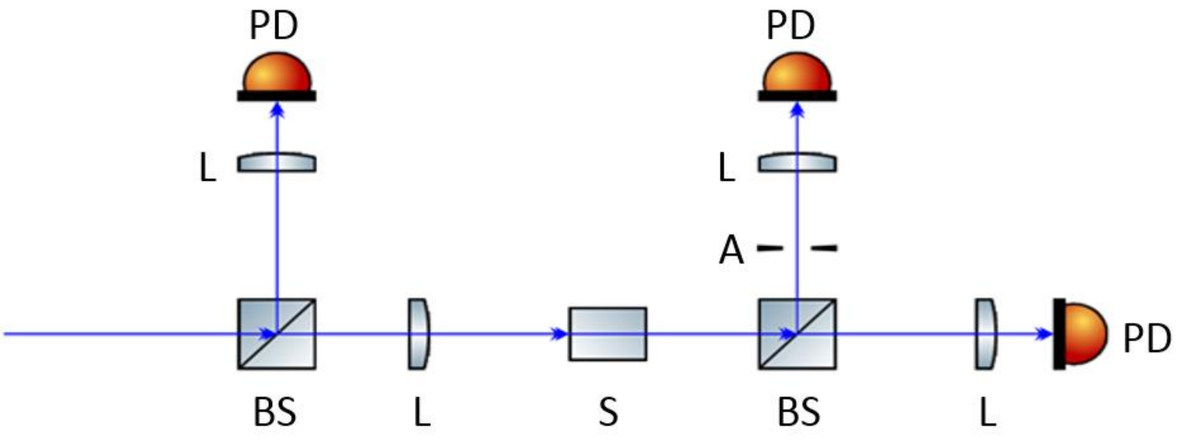

Scheme S1 *Z-scan setup whereby BS is a beam splitter, L is lens, PD – a photodiode, S – a sample, A – an aperture for the closed aperture z-scans. The blue line is the laser beam used for excitation.*

Wide angle x-ray scattering (WAXS): In order to perform x-ray scattering measurements, the sample has been dried into a film as follows: 10 mL of the 2% concentrated fibril solution have been evaporated on Teflon tape, 1 mL per time. This method applies for both the thermically treated and the original fibril solution. Synchrotron SAXS measurements were performed at the Swiss Light Source, cSAXS, Paul Scherrer Institute, at a wavelength of 1 Å and detector distance of 0.236 m. The X-ray beam was focused on the detector to 51 × 17 μm^2 (horizontal x vertical), and impinged parallelly to the surface of the films, for both the standard amyloids fibrils and for the hydrothermally treated ones. Scanning-WAXS[3] images were collected with a pixel size of 50 µm, and the resulting signals were averaged in Fig.1 over 1476 WAXS patterns per sample in the areas shown in Fig. Sx. The measurement intensity was corrected by the transmission, which corrects also for sample thickness. All the codes were developed by the Coherent X-ray Scattering group at the Paul Scherrer Institute in Villigen, Switzerland and can be found on the cSAXS web page at https://www.psi.ch/sls/csaxs/software.

Supplementary Figures

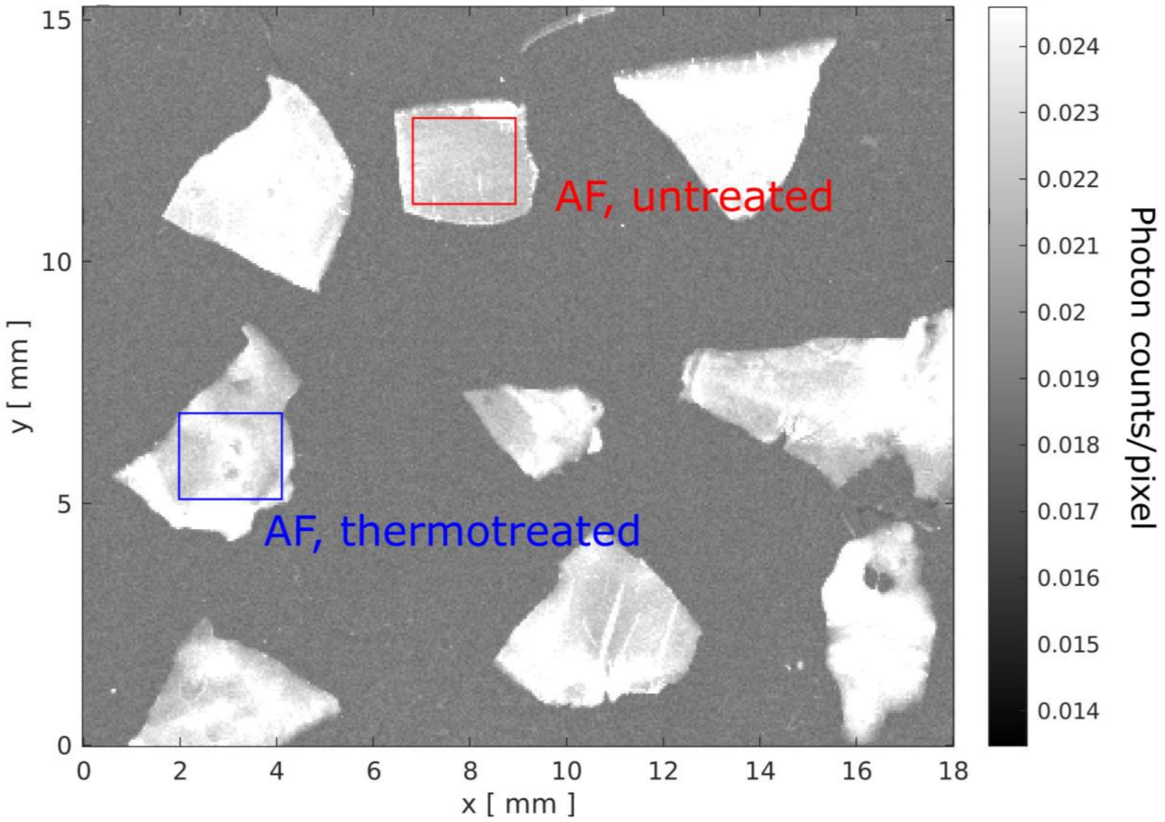

Fig. S1 Scanning-WAXS images of untreated and thermotreated amyloid fibrils. The color bar indicates the scattering intensity (photon counts per pixel) of the films in the range of 4 - 6 Å. The selected ROI (region of interest) covers about 41 x 36 pixels, which results in the average among 1476 scattering patterns per sample.

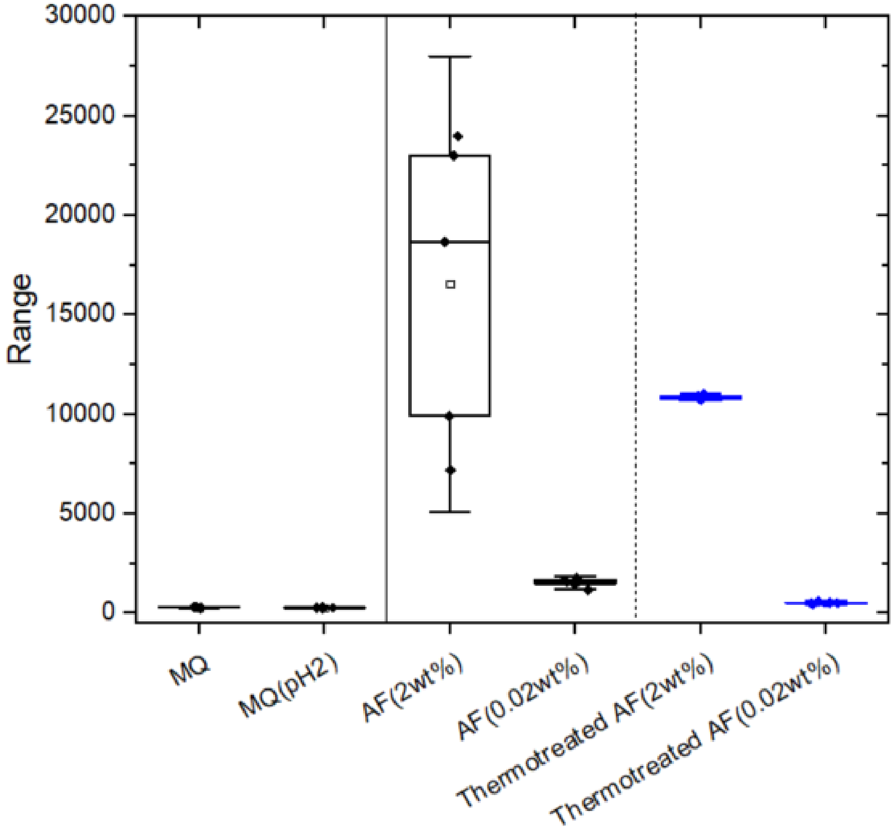

Fig. S2 ThT fluorescence assay showing the abundance of β-sheet content of amyloid fibril solution before (**1**) and after (**2**) hydrothermal treatment at the concentration of . Error bars represent mean ± S.D.

The acquisition of IR spectra aimed to detect the presence of β-sheets in the hydrothermally treated β-lactoglobulin fibrils (Figure S1, left panel). The absence of a peak at 1611-1630 cm^{-1} suggests that the β-sheets structure is not retained and the random coil population is dominant[4].

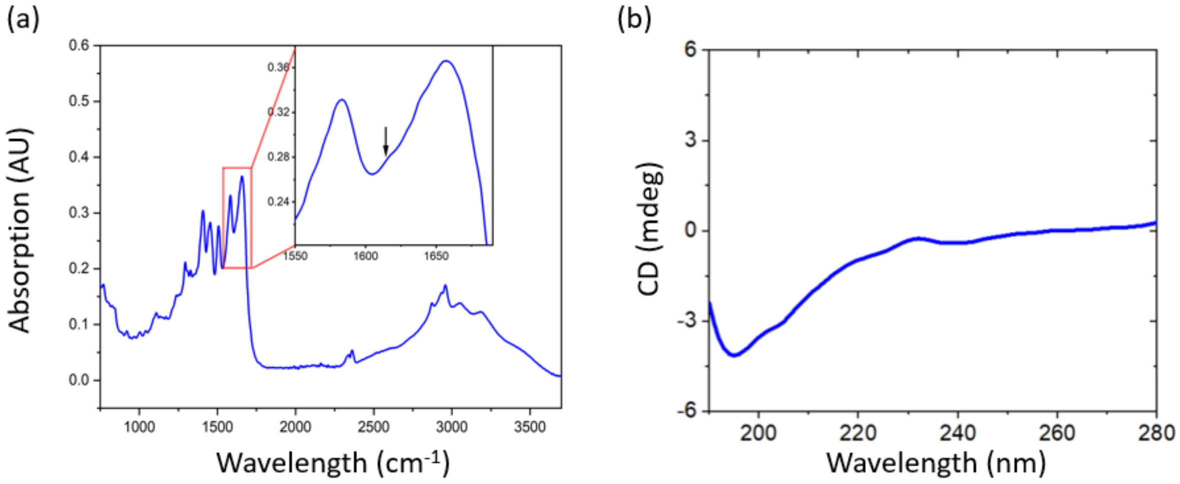

Fig. S3 FTIR and CD spectra of the hydrothermally treated β-lactoglobulin fibrils (**1**).

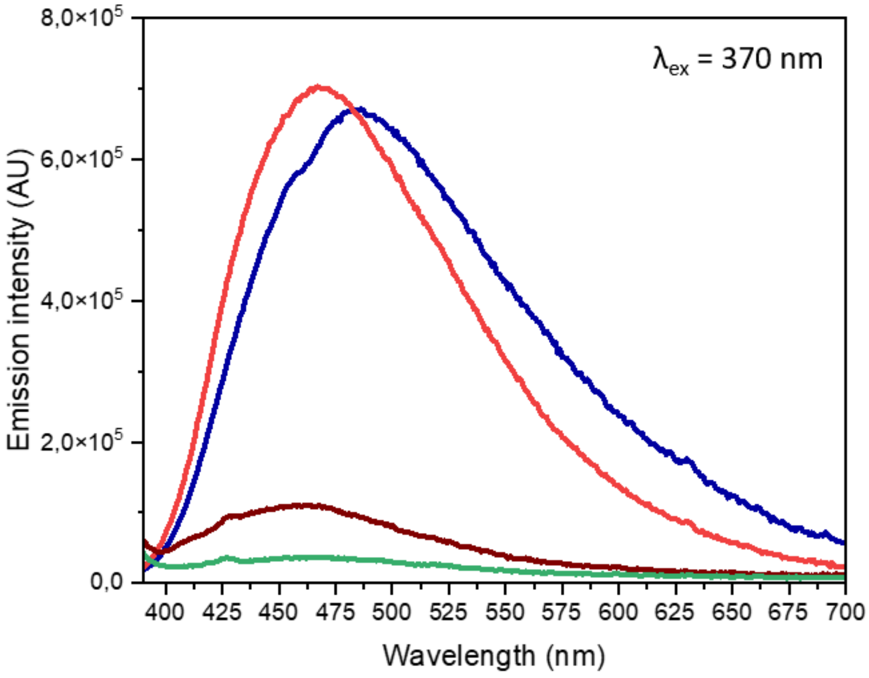

Fig. S4 Fluorescence spectra of hydrothermally treated β-lactoglobulin fibrils (**1**) (dark blue), standard β-lactoglobulin fibrils (**2**) (brown), hydrothermally treated protein monomers (**3**) (red), and untreated monomers (green).

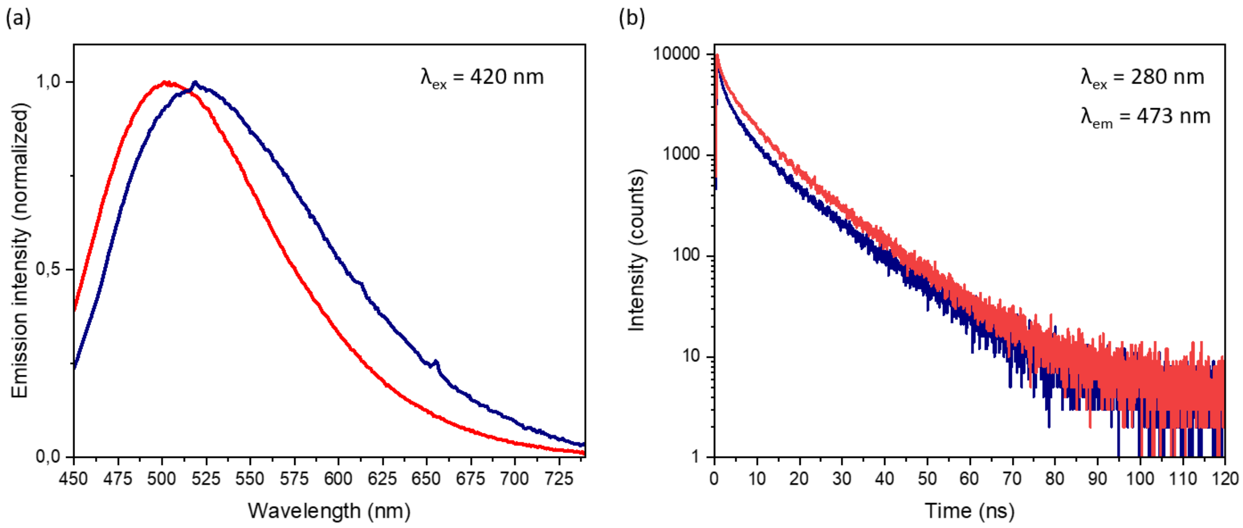

Fig. S5 Fluorescence spectra and fluorescence lifetimes of the hydrothermally treated β-lactoglobulin fibrils (**1**) (blue) and hydrothermally treated β-lactoglobulin monomers (**3**) (red).

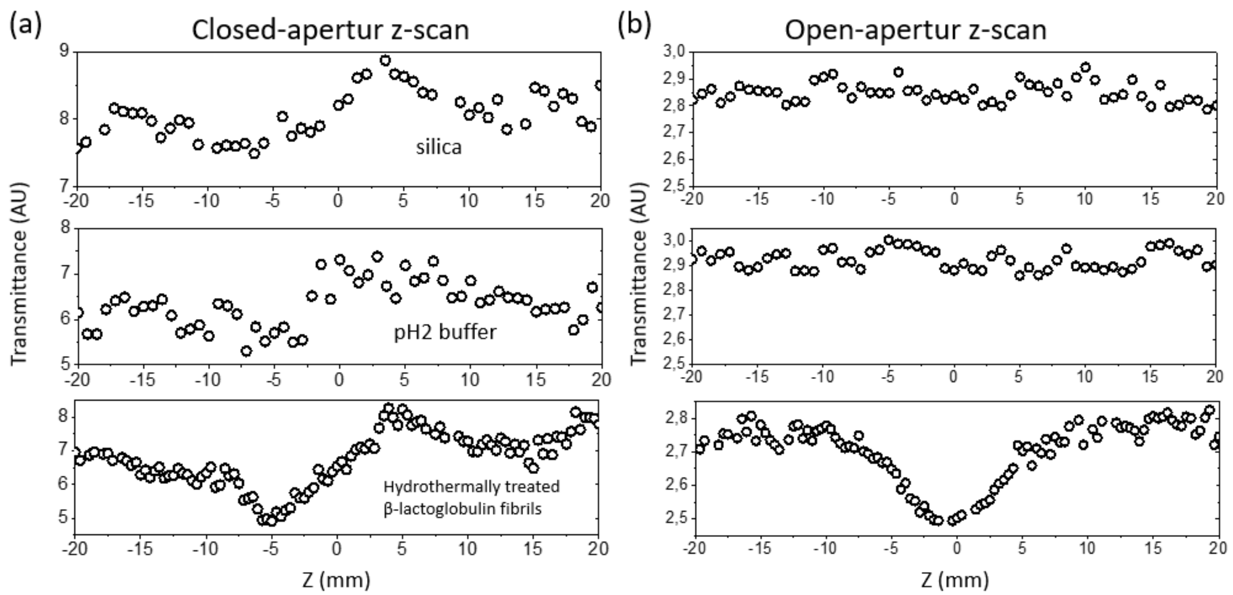

Fig. S6 Example of single experiment whereby closed aperture (CA) (left panel) and open aperture (OA) (right panel) z-scan was calibrated on silica and buffer pH2 to avoid absorption from glass and solvent and measurement of CA and OA in β-lactoglobulin fibrils (**1**) after hydrothermal treatment at 510 nm.

Quantum chemical calculations: To understand the experimentally observed enhancement of 2PA in β-lactoglobulin and to find the underlying mechanism responsible for it, quantum chemical calculation were performed using dimers of the chromophore which is structurally referring to aromatic residue - tyrosine. The calculation was performed at different intermolecular distances. The electronic structure response theory within the framework of time-dependent density functional theory (TD-DFT) was used to examine the influence of intermolecular distance in the tyrosine dimer on respective 2PA cross-sections. All TD-DFT calculations were performed using the CAM-B3LYP functional[5]. At both levels, the cc-pVDZ basis set[6] was used and 15 singlet excited states were considered (Fig. S3).

Model systems were created from the peptide fibril crystal structure 3HYD[7]. 8 heavy atoms from the tyrosine side chain (beta carbon, six carbons in aromatic ring and phenol oxygen) were extracted from the crystallographic structure. Hydrogen atoms (eight in total) were then added using the Avogadro program package[8]. Thus, the molecule 4-methylphenol is used as a model for tyrosine. All the data in tabular form has been provided table S1 and S2.

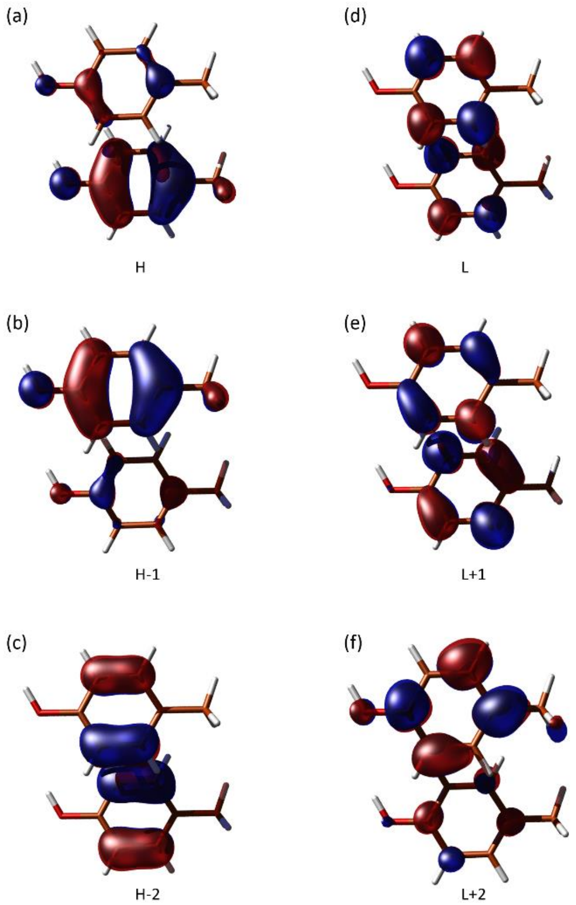

Fig. S7 *Calculations were carried out with cc-pVDZ basis set (giving 58 occupied orbitals for the dimer system). 15 excitations were calculated and analysed for the dimers and 6 representative are shown. Orbitals were*

visualized with the Molden program for analysis using a contour value of 0.05.

Table S1: CC2/cc-pVDZ

Excitation wavelength	Photon wavelength	Oscillator strength	2PA transition strength	orbitals
247.1 nm	494.2 nm	0.073	6	H -> L
244.9 nm	489.8 nm	0.003	6	H-1->L
194.3 nm	388.6 nm	0.012	11	H-2->L, H->L+2
192.2 nm	384.3 nm	0.210	114	H-1->L+2
180.7 nm	361.3 nm	0.037	113	H ->L
177.8 nm	355.6 nm	0.034	80	H-2->L
175.9 nm	351.9 nm	0.017	38	H-1->L+5

Table S2: CC2/aug-cc-pVDZ

Excitation wavelength	Photon wavelength	Oscillator strength	2PA transition strength	orbitals
256.2 nm	512.4 nm	0.068	8	H ->L+16
253.8 nm	507.7 nm	0.001	8	H-1->L+16
240.5 nm	481.0 nm	0.000	8	H-1->L
233.6 nm	467.2 nm	0.001	6	H ->L+2
222.1 nm	444.2 nm	0.000	3	H ->L+1
218.4 nm	436.8 nm	0.001	3	H-1->L+1
212.1 nm	424.2 nm	0.024	28	H ->L+3

Important to note is that the maximum 2PA in calculations at 380 nm does not exactly match with the experiment which was performed down to the technical limitation of the z-scan experiment at 390 nm. Many factors may contribute to this discrepancy. In particular, the theoretical calculations were performed in the vacuum phase. TD-DFT/CAM-B3LYP is also known to overestimate the excitation energies and it usually requires spectral shifting when compared with the experimental data[9]. In the case of similar studies in YFP system the shift was as large as +46 nm[10].

Direct comparison of the ICT transitions found in this work is not possible because the calculations do not include the explicit effect of the protein environment and the experimental spectra do not shed light on the nature of the excitations. The calculations confirm only the experimental studies that 2PA enhancement is strongly affected by the ICT transitions and that the distance between aromatic residues as well as their number is directly involved in the yield of the 2PA.

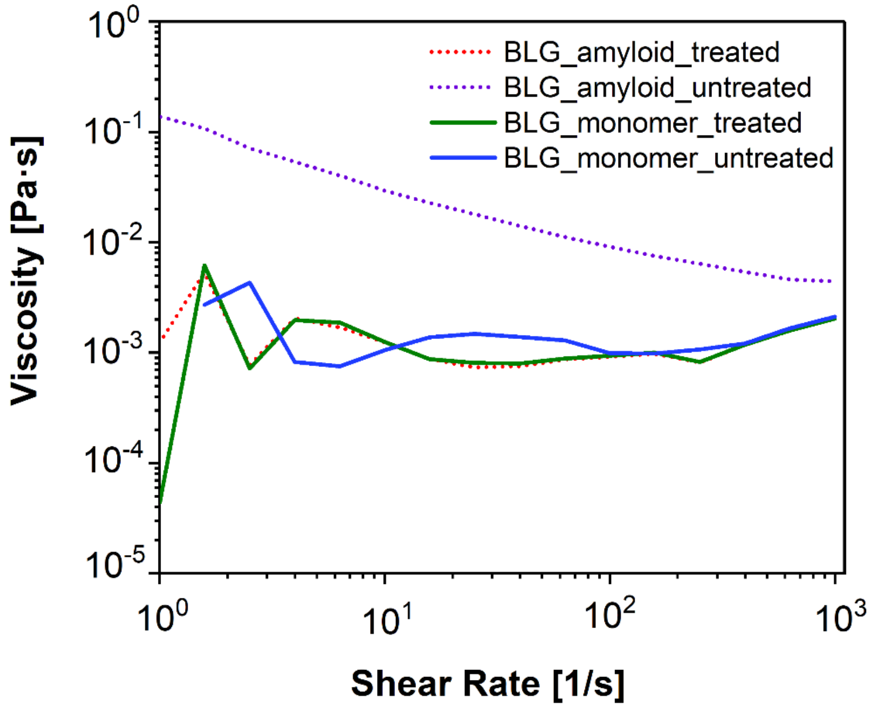

Fig. S8 *Shear rheology results on β-lactoglobulin protein materials. Hydrothermally treated fibrils show lower viscosity than untreated fibrils.*

Multi-Speckle Diffusing-Wave Spectroscopy (MS-DWS) (Rheolaser): The microrheological properties of hydrothermally treated β-lactoglobulin fibrils and hydrothermally treated β-lactoglobulin monomers were measured using the Multi-Speckle Diffusing-Wave Spectroscopy (MS-DWS) technique (Rheolaser MASTER™, Formulaction, France), according to the patented algorithm[11]. MS-DWS operates by transmitting a coherent laser beam ($\lambda = 650$ nm) into the sample, which interacts with particles and results in a speckle pattern due to wave interference, captured by a multi-pixel (CCD) camera detector. These speckle patterns, indicative of particle Brownian motion, reflect the particles velocity and the extent of their movement. In viscoelastic samples, the scatterers, being mobile due to Brownian motion, cause fluctuations in light intensity in the speckle image, leading to deformation of the overall speckle pattern. The temporal evolution of the speckle image provides insights into the material's viscoelastic properties. The samples (4 mL) were transferred into flat-bottomed glass vials and placed in the instrument sample-holder, and backscattered light intensity was detected at 20 °C for 30 minutes. The microrheological parameters, i.e., Mean Square Displacement (MSD value is the average of a number of scattering trajectory where the unit is run)[12], Elasticity Index (EI value is reciprocal of the MSD value on the plateau of the curve in the middle decorrelation time), Macroscopic Viscosity Index (MVI value is reciprocal of the MSD value in the long decorrelation time), were collected and processed by a RheoSoft MASTER[13].

Figure S9 illustrates the measurements of the Brownian motion of the particle as the particle Mean Square Displacement (MSD) as a function of time. The relatively linear evolution of the MSD plots indicates that

liquid-like behavior dominated in the thermotreated samples. However, the shift of the curve for fibrils **(3)** towards a lower MSD level and longer decorrelation time, compared to the curve for monomers **(1)**, demonstrates stronger elastic intermolecular interactions and an increase in the macroscopic viscosity of the formulation with fibrils. This is reflected in the significant differences in MVI and EI values, computed for the two thermally treated forms of β-lactoglobulin (as shown in Table S3). The higher viscosity and elasticity of the hydrothermally treated fibrils were beneficial when processing the materials into macroscopic forms, such as gels.

Tab. S3 Viscosity and elasticity parameters of thermotreated β-lactoglobulin

	Hydrothermally treated β-lactoglobulin fibrils (**1**)	Hydrothermally treated β-lactoglobulin monomers (**3**)
Macroscopic viscosity index (MVI) (× 10^4 nm^{-2})	6.63 ± 0.97	1.21 ± 0.30
Elasticity Index (EI) (× 10^3 nm^{-2})	2.78 ± 0.12	1.81 ± 0.04

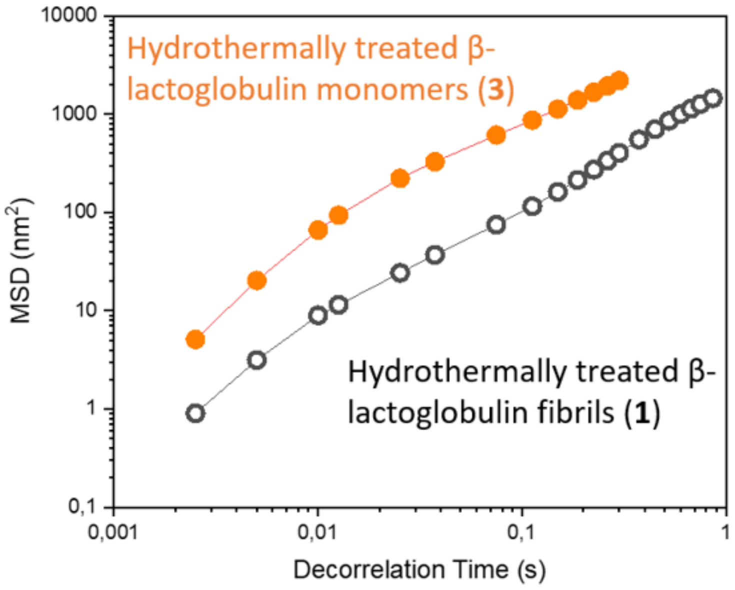

Fig. S9 Rheolaser, leveraging Diffusing Wave Spectroscopy data displayed as particle Mean Square Displacement (MSD) versus time.

References

1. Jung, J.-M.; Savin, G.; Pouzot, M.; Schmitt, C.; Mezzenga, R., Structure of heat-induced β-lactoglobulin aggregates and their complexes with sodium-dodecyl sulfate. *Biomacromolecules* **2008,** *9* (9), 2477-2486.
2. Sheik-Bahae, M.; Said, A. A.; Wei, T.-H.; Hagan, D. J.; Van Stryland, E. W., Sensitive measurement of optical nonlinearities using a single beam. *IEEE journal of quantum electronics* **1990,** *26* (4), 760-769.
3. Eichler, R. K., D.; Koschik, A.; Knecht, A.; van der Meulen, N.; Scheibl, R.; Barth, L.; Baumgarten, C.; Caminada, L.; Chang, J.; et al. , IMPACT conceptual design report. *(PSI Bericht, Report No.: 22-01). Paul Scherrer Institut.* **2022**, 302.
4. Zandomeneghi, G.; Krebs, M. R.; McCammon, M. G.; Fändrich, M., FTIR reveals structural differences between native β-sheet proteins and amyloid fibrils. *Protein science* **2004,** *13* (12), 3314-3321.
5. Yanai, T.; Tew, D. P.; Handy, N. C., A new hybrid exchange–correlation functional using the Coulomb-attenuating method (CAM-B3LYP). *Chemical physics letters* **2004,** *393* (1-3), 51-57.
6. Dunning Jr, T. H., Gaussian basis sets for use in correlated molecular calculations. I. The atoms boron through neon and hydrogen. *The Journal of chemical physics* **1989,** *90* (2), 1007-1023.
7. Ivanova, M. I.; Sievers, S. A.; Sawaya, M. R.; Wall, J. S.; Eisenberg, D., Molecular basis for insulin fibril assembly. *Proceedings of the National Academy of Sciences* **2009,** *106* (45), 18990-18995.
8. Hanwell, M. D.; Curtis, D. E.; Lonie, D. C.; Vandermeersch, T.; Zurek, E.; Hutchison, G. R., Avogadro: an advanced semantic chemical editor, visualization, and analysis platform. *Journal of cheminformatics* **2012,** *4* (1), 1-17.
9. Beerepoot, M. T.; Steindal, A. H.; Kongsted, J.; Brandsdal, B. O.; Frediani, L.; Ruud, K.; Olsen, J. M. H., A polarizable embedding DFT study of one-photon absorption in fluorescent proteins. *Physical Chemistry Chemical Physics* **2013,** *15* (13), 4735-4743.
10. Beerepoot, M.; Friese, D.; Ruud, K., Intermolecular charge transfer enhances two-photon absorption in yellow fluorescent protein. *Physical Chemistry Chemical Physics* **2014,** *16* (13), 5958-5964.
11. Degrand, L.; Michon, C.; Bosc, V., New insights into the study of the destabilization of oil-in-water emulsions with dextran sulfate provided by the use of light scattering methods. *Food Hydrocolloids* **2016,** *52*, 848-856.
12. Cristiano, M. C.; Froiio, F.; Mancuso, A.; De Gaetano, F.; Ventura, C. A.; Fresta, M.; Paolino, D., The Rheolaser Master™ and Kinexus rotational rheometer® to evaluate the influence of topical drug delivery systems on rheological features of topical poloxamer gel. *Molecules* **2020,** *25* (8), 1979.
13. Larsen, T. H.; Furst, E. M., Microrheology of the liquid-solid transition during gelation. *Physical review letters* **2008,** *100* (14), 146001.
